# Incretin-Modulated Beta Cell Energetics in Intact Islets of Langerhans

**DOI:** 10.1210/me.2014-1038

**Published:** 2014-04-25

**Authors:** David J. Hodson, Andrei I. Tarasov, Silvia Gimeno Brias, Ryan K. Mitchell, Natalie R. Johnston, Shahab Haghollahi, Matthew C. Cane, Marco Bugliani, Piero Marchetti, Domenico Bosco, Paul R. Johnson, Stephen J. Hughes, Guy A. Rutter

**Affiliations:** Section of Cell Biology, Division of Diabetes, Endocrinology and Metabolism, Department of Medicine (D.J.H, A.I.T., S.G.B., R.K.M., N.R.J., S.H., M.C.C., G.A.R.), Imperial College London, London W12 0NN, United Kingdom; Department of Endocrinology and Metabolism (M.B., P.M.), University of Pisa, 56126 Pisa, Italy; Cell Isolation and Transplantation Center, Department of Surgery (D.B.), Geneva University Hospitals and University of Geneva, 1205 Geneva, Switzerland; Oxford Centre for Diabetes, Endocrinology, & Metabolism (P.R.J., S.J.H.), University of Oxford, Oxford OX3 7LE, United Kingdom; NIHR Oxford Biomedical Research Centre (P.R.J., S.J.H.), Churchill Hospital, Oxford OX3 7LE, United Kingdom; and Nuffield Department of Surgical Sciences (P.R.J., S.J.H.), University of Oxford, Oxford OX3 9DU, United Kingdom

## Abstract

Incretins such as glucagon-like peptide 1 (GLP-1) are released from the gut and potentiate insulin release in a glucose-dependent manner. Although this action is generally believed to hinge on cAMP and protein kinase A signaling, up-regulated beta cell intermediary metabolism may also play a role in incretin-stimulated insulin secretion. By employing recombinant probes to image ATP dynamically in situ within intact mouse and human islets, we sought to clarify the role of GLP-1-modulated energetics in beta cell function. Using these techniques, we show that GLP-1 engages a metabolically coupled subnetwork of beta cells to increase cytosolic ATP levels, an action independent of prevailing energy status. We further demonstrate that the effects of GLP-1 are accompanied by alterations in the mitochondrial inner membrane potential and, at elevated glucose concentration, depend upon GLP-1 receptor-directed calcium influx through voltage-dependent calcium channels. Lastly, and highlighting critical species differences, beta cells within mouse but not human islets respond coordinately to incretin stimulation. Together, these findings suggest that GLP-1 alters beta cell intermediary metabolism to influence ATP dynamics in a species-specific manner, and this may contribute to divergent regulation of the incretin-axis in rodents and man.

Type 2 diabetes is a socioeconomically costly disease state usually characterized by pancreatic beta cell decompensation in the face of increased resistance to circulating insulin (1). The resulting glucose intolerance leads to undesirable sequelae including neuropathy, renal failure, cardiac disease, and increased cancer risk. Under normal conditions, the secretion of insulin is primarily driven by the aerobic glycolysis of glucose, raising cytosolic ATP/ADP ratios [ATP/ADP]_cyto_. This leads to the closure of hyperpolarizing ATP-sensitive K^+^ channels (K_ATP_) and calcium (Ca^2+^)-dependent exocytosis due to Ca^2+^ influx through voltage-dependent Ca^2+^ channels (VDCC). Secretion is further augmented by “amplifying” pathways (2, 3), which may involve intracellular signaling cascades such as those mediated by cAMP, acting upstream of exchange protein activated by cAMP (Epac) (4) and protein kinase A (5), as well as AMP-activated protein kinase (6), protein kinase C (7) and MAPK (8, 9).

In addition to glucose, a number of alternative fuels and circulating factors regulate insulin secretion. Notably, gut-derived incretins including glucagon-like peptide 1 (GLP-1) and glucose-dependent insulinotropic polypeptide are liberated from entero-endocrine cells in response to bile acid and nutrient flux (10, 11), and act to potentiate insulin release in a glucose-dependent manner (12, 13). Due to the latter and other properties, incretin-based analogs are becoming mainstays of type 2 diabetes treatment. Although the effects of GLP-1 upon adenylate cyclase (AC) activity, [cAMP]_i_ oscillations, Epac signaling and exocytosis are increasingly well characterized (4, 14), whether incretins are able to alter beta cell intermediary metabolism to influence insulin secretion remains controversial. Thus, whereas dynamic luciferase-based studies by us have demonstrated increased free cytosolic [ATP] in GLP-1-stimulated MIN6 immortalized beta cells (15), others have failed to detect any effect of the GLP-1 mimetic, exendin-4, on mitochondrial ATP levels in primary rodent islets (16). Nonetheless, the latter studies did report a significant increase in glucose utilization in response to GLP-1 at elevated glucose concentration, although glucose oxidation was not changed by the incretin at the time points studied.

Further suggesting that incretin-stimulated insulin secretion may in part involve altered metabolism are the observations that: 1) GLP-1 and exendin-4 stimulate large oscillations in intracellular Ca^2+^ concentrations ([Ca^2+^]_i_), both in rodent and human beta cells (16–18); 2) ATP-consuming processes are required for cytoplasmic Ca^2+^ removal and intracellular store refilling (19); 3) mitochondrial Ca^2+^ uptake activates citrate cycle dehydrogenases, augmenting ATP production (20); 4) excessive mitochondrial Ca^2+^ uptake may depolarize the inner mitochondrial membrane, resulting in a temporary cessation of ATP synthesis (21); and 5) Ca^2+^ stimulates energy-consuming processes such as exocytosis (22). Correspondingly, we (19) and others (21) have recently demonstrated that glucose-dependent oscillations in intracellular ATP are strongly influenced by Ca^2+^ in pancreatic beta cells.

To further investigate a role for incretin in beta cell energetics, we used a recombinant strategy to direct expression of the GFP-based ATP-binding protein Perceval (23) throughout the first few layers of mouse and human primary islets (21). Although previously deployed in dissociated beta cells (24–26) and small numbers of cells in intact islets (21), this technique has not been employed to investigate ATP/ADP dynamics across a large population, nor have responses to incretins been examined in islets.

Using this approach, we show that GLP-1 modulates ATP dynamics, resulting in [ATP/ADP]_cyto_ rises. These effects were independent of prevailing cell metabolic status because GLP-1 was still able to induce ATP oscillations in the presence of low (3 mM) glucose concentration, which is nonpermissive for insulin secretion. Moreover, GLP-1-stimulated ATP dynamics at elevated glucose concentration were reliant upon engagement of the GLP-1-receptor (GLP-1R) and ensuing Ca^2+^ influx because they could be blocked reversibly using exendin 9-39 and verapamil, respectively. Lastly, species differences in GLP-1-regulated “metabolic connectivity” were present, with mouse but not human islets responding to stimulus with synchronous ATP dynamics. Thus, GLP-1 affects beta cell intermediary metabolism through alterations to ATP dynamics in a species-specific manner, and this may play an important role in incretin-modulated beta cell function at both low and elevated glucose concentrations.

## Results

### Adenovirus-harboring Perceval is tropic for beta cells and reports ATP changes in intact islets

**Figure 1.**
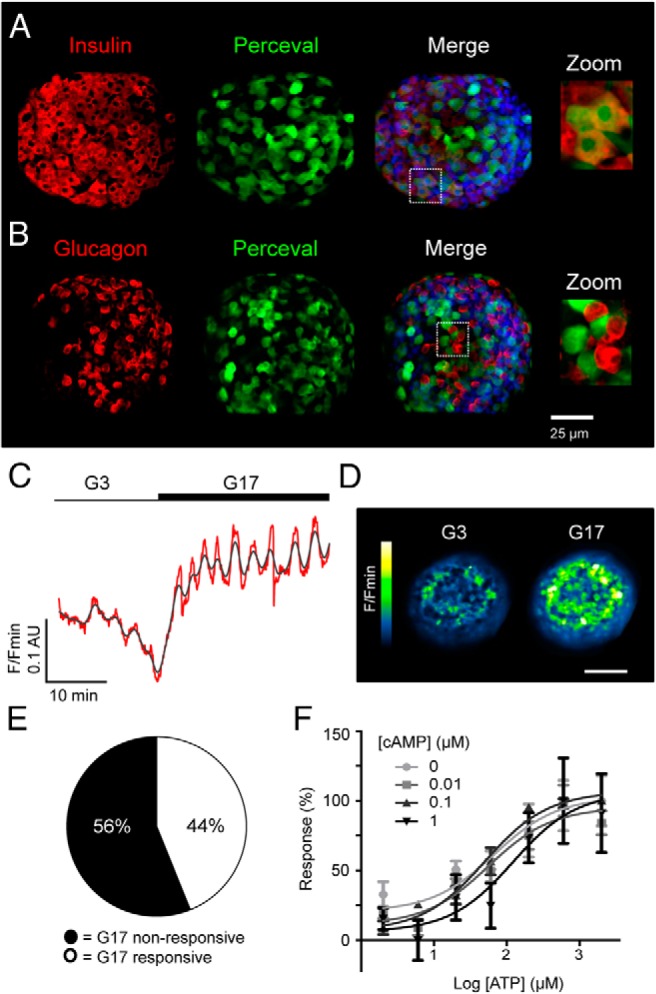
The ATP/ADP-sensor Perceval reports beta cell metabolism in intact islets. A, Specific immunohistochemistry for insulin demonstrates the preferential expression of Perceval in beta cells (DAPI, blue; scale bar shown). B, As for A, but immunostaining against glucagon showing absence of the probe in alpha cells. C, Elevated glucose concentration increases Perceval fluorescence reflecting increases in cytoplasmic ATP/ADP ratio ([ATP/ADP]_cyto_). D, Representative recording of a Perceval-expressing islet before and during exposure to high glucose (G17; 17 mM glucose) (n = 14 recordings from six animals) (red, raw; gray, smoothed) (scale bar, 50 μm). E, Glucose engages a subpopulation (44%) of metabolically-active beta cells, which respond with [ATP/ADP]_cyto_ increases. F, Dose-response graph depicting ATP-induced increases in dialyzed Perceval fluorescence, which are unaffected by the presence of increasing cAMP concentrations (0–1 μM) (sigmoidal dose-response fitted to 2–3 independent repeats).

Specific immunohistochemical analyses detected Perceval expression predominantly in the insulin immunopositive cell population, confirming the reported (27–29) tropism of adenoviruses for beta (Figure 1A) over alpha (Figure 1B) and other endocrine cells within mouse islets of Langerhans. Nipkow spinning disk microscopy (30) was therefore used to capture the effects of glucose and other secretagogues on cytoplasmic ATP/ADP dynamics throughout the population of beta cells residing within the first few layers of intact islets. Elevation of glucose from 3–17 mM induced a multiphasic response typified by an initial increase in [ATP/ADP]_cyto_ (F/Fmin), followed by the development of superimposed oscillations, as previously reported (Figure 1, C and D) (ΔATP/ADP = 0.13 ± 0.02 AU, n = 14 recordings from six animals) (21). Just under half of the imaged population (44%) responded to glucose with increases in apparent [ATP]_cyto_ (Figure 1E), suggesting that a subpopulation of beta cells may act to integrate metabolic information before propagating this throughout the syncytium as Ca^2+^ waves (31, 32).

To determine whether cAMP could interfere with the Perceval ATP-binding site, HEK293 cells expressing the ATP sensor were lysed before dialysis and exposure to cAMP at varying concentrations of ATP. Consistent with our previous studies (25, 26), Perceval displayed a ∼10–30% increase in fluorescence intensity following ATP binding in the absence of ADP, and this was similar following incubation with increasing concentrations of cAMP (0.1–1 μM), (logEC50 = 1.83, 1.75, 1.70, and 2.1 at 0, 0.01, 0.1, and 1 μM cAMP, respectively) (Figure 1F).

### GLP-1 induces oscillations in cytosolic ATP/ADP

**Figure 2.**
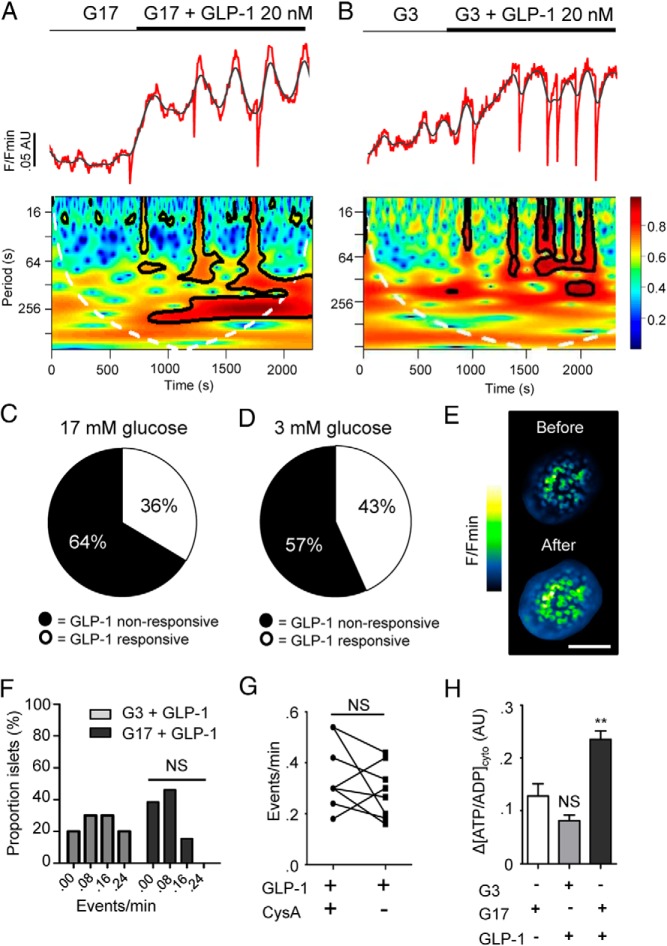
GLP-1 induces [ATP/ADP]_cyto_ increases in intact islets under low and high glucose conditions. A, 20 nM GLP-1 increases cytoplasmic ATP in islets perifused with 17 mM glucose (G17) (top panel; representative trace; red, raw; gray, smoothed). Wavelet analysis shows the effects of GLP-1 on the period and power (0–1 = blue-red) of ATP oscillation frequency. B, As for A, but islets exposed to 3 mM glucose (G3). C, GLP-1 engages a subpopulation of metabolically-coupled beta cells at 17 mM glucose. D, As for C but in the presence of 3 mM glucose. E, Representative images showing Perceval fluorescence in an islet before and after introduction of GLP-1 (image cropped to display a single islet; scale bar, 115 μm). F, Glucose concentration does not modulate the effects of GLP-1 on ATP oscillation frequency (NS, nonsignificant; Mann-Whitney *U* test on the nonbinned data, n = 13 recordings from six animals). G, Cyclosporin A (CysA) does not prevent appearance of downward deflections in [ATP/ADP]_cyto_ (NS, nonsignificant vs GLP-1 + CysA; Student paired *t* test, n = 8 recordings from four animals). H, Glucose and GLP-1 are equipotent at elevating [ATP/ADP]_cyto_ in islets and the incretin can elicit additional increases at high (17 mM) glucose concentration (NS, nonsignificant and **, *P* < .01 vs G3; one-way ANOVA followed by Bonferonni's post hoc test, n = 13–14 recordings from six animals).

In the presence of high (17 mM) glucose concentration, the addition of 20 nM GLP-1 to Perceval-expressing mouse islets stimulated increases in fluorescence, which were superseded by the appearance of low-frequency oscillations (Figure 2A). This dose of incretin has previously been shown to maximally stimulate intracellular Ca^2+^ rises in islets (17). Importantly, in our hands, GLP-1 evoked a barely detectable decrease in intracellular pH (∼0.05 pH unit) (23, 26), indicating that the fluctuations in Perceval fluorescence intensity are likely to result from oscillations in [ATP/ADP]_cyto_, as opposed to acidification/alkalinization. Following incubation at nonpermissive (3 mM) glucose concentration, GLP-1 was similarly able to induce oscillatory increases in Perceval fluorescence (Figure 2B), although the incretin was unable to alter Ca^2+^ responses under these conditions (81.1 ± 6.0 vs 8.9 ± 2.3% GLP-1-responsive cells, G3 + GLP-1 vs G11 + GLP-1, respectively; n = 6 recordings from three animals, *P* < .01). As observed for the effects of glucose (Figure 1E), the action of GLP-1 involved the recruitment of a subnetwork of beta cells. Thus, ∼40% of the recorded population responded to GLP-1 in the above manner (Figure 2, C—E). Intriguingly, sharp drops in apparent [ATP/ADP]_cyto_ were seen between peaks (Figure 2, A and B). These deflections seemed to be part of normal stochastic behavior because they were not influenced by prevailing glucose concentration (Figure 2F) and persisted in the presence of cyclosporin A (CysA), an inhibitor of the mitochondrial permeability transition pore (Figure 2G) (33). Although GLP-1 tended to stimulate smaller [ATP/ADP]_cyto_ rises than 17 mM glucose, this was not significant (ΔATP/ADP = 0.13 ± 0.02 vs 0.08 ± 0.01 AU, 17 mM glucose vs GLP-1 applied at 3 mM glucose, respectively; n = 13–14 recordings from six animals, nonsignificant, NS) (Figure 2H). Notably, the incretin was still able to augment [ATP]_cyto_ even in the continued presence of high (17 mM) glucose (Figure 2H). Similar results were obtained using conventional luciferase-based detection of ATP (Table 1).

**Table 1.** Luciferase-Based Measures of ATP Concentration in Glucose- and GLP-1-Treated Islets (n = 8 animals)

GLP-1 nm Glucose mm	0		20	
	3	17	3	17
ATP pmol/islet	232.7 ± 50.2	495.9 ± 105.7^a^	797.5 ± 230.3^b^	1002 ± 183.2^b^

a *P* < .05 vs 3 mm glucose-alone; Student *t* test.

b *P* < .05 vs 3 mm glucose-alone; ANOVA followed by Dunnett's multiple comparison test.

### GLP-1 drives increases in inner mitochondrial membrane potential, which are dependent on glucose

At elevated glucose concentration, both enhanced supply of glycolytically derived pyruvate and Ca^2+^ activation of key dehydrogenases (20) accelerate citrate cycle flux in beta cell mitochondria, elevating the inner mitochondrial membrane potential (ΔΨ_m_) (26). The consequent activation of the F_1_/F_0_ ATP-synthase then results in accelerated ATP production, potentially raising [ATP/ADP]_cyto_ and countering decreases in the latter driven by cytosolic ATP-consuming reactions. Conversely, excessive Ca^2+^ uptake, and hence an accumulation of positive charge, may exert a direct depolarizing effect to lower ΔΨ_m._

**Figure 3.**
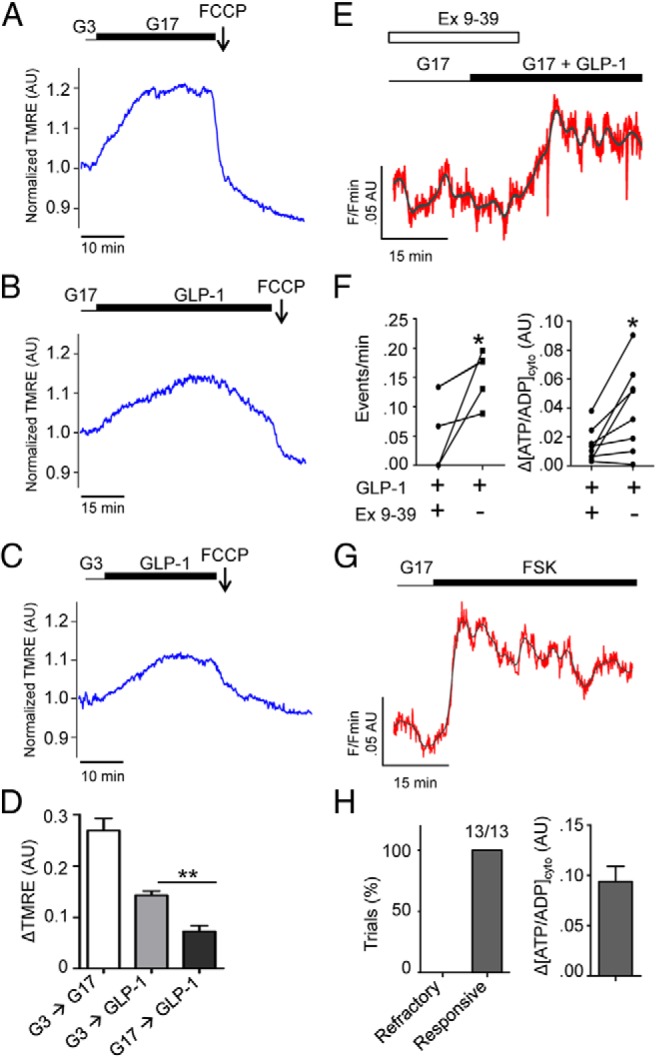
GLP-1 decreases mitochondrial potential and engages its cognate receptor to increase [ATP/ADP]_cyto_. A, Elevated glucose (17 mM; G17) stimulates rapid and large excursions in TMRE fluorescence, indicating mitochondrial hyperpolarisation (representative trace from n = 6 recordings from four animals). B, GLP-1 under elevated glucose concentration slowly and subtly increases TMRE fluorescence, indicating minor hyperpolarizing effects on mitochondrial potential (representative trace from n = 6 recordings from four animals). C, As for B but in the presence of 3 mM glucose (representative trace from n = 7 recordings from four animals). D, TMRE responses to GLP-1 are significantly lower than those to glucose (**, *P* < .01 vs G17-alone, one-way ANOVA followed by Bonferonni's post hoc test). E, Representative Perceval trace showing reversible blockade of GLP-1 effects by 100 nM exendin 9-39 (Ex 9-39) (gray, raw; red, smoothed). F, Ex 9-39 abolishes GLP-1-induced [ATP/ADP]_cyto_ dynamics and rises (*, *P* < .05 vs GLP-1 + Ex 9-39, Student paired *t* test, n = 11 recordings). G, Forced elevations in cAMP using forskolin (FSK) lead to increases in [ATP/ADP]_cyto_. H, FSK evoked ATP rises in all experiments performed (13/13). Unless otherwise stated, experiments are from islets taken from ≥ three animals.

To determine whether GLP-1-induced increases in [ATP/ADP]_cyto_ may be due at least in part to increases in Ψ_m_, we dynamically tracked fluorescence of the potential-sensitive fluorescent probe tetramethyl rhodamine ethylester (TMRE) in beta cells within whole islets. In response to elevated (17 mM) glucose, cells demonstrated rapid and large increases in TMRE intensity, indicative of mitochondrial hyperpolarization (Figure 3A), as expected (19, 34, 35). By comparison, GLP-1 at both low (3 mM) and high (17 mM) glucose concentrations induced slower and smaller increases in TMRE fluorescence (Figure 3, B and C). This observation is concordant with the smaller [ATP/ADP]_cyto_ increase elicited by GLP-1 vs glucose. However, the smaller increase in ΔΨ_m_ elicited by GLP-1 at 17 mM compared with 3 mM glucose (∼0.1 AU) is discordant with the larger increment change in [ATP/ADP]_cyto_ induced by the incretin at the same glucose concentration. Hence, GLP-1 causes a larger increase in [ATP/ADP]_cyto_ for a given ΔΨ_m_ increase at high (17 mM) glucose concentration (Figure 3D).

### Cytosolic ATP/ADP increases require GLP-1 receptor activation

We next sought to determine whether GLP1-R occupancy was a prerequisite for the modulation of ATP dynamics by GLP-1. Confirming a role for the GLP-1R in beta cell metabolism was the finding that exendin 9-39 (Ex 9-39), a specific GLP-1R antagonist (36), reversibly prevented GLP-1 from affecting ATP dynamics (0.08 ± 0.02 vs 0.14 ± 0.02 events/minutes, during and after Ex 9-39, respectively; n = 8 recordings, *P* < .05) (Figure 3, E and F).

To assess whether the effects of GLP-1 on [ATP/ADP]_cyto_ were likely to involve the generation of intracellular cAMP, islets were exposed to forskolin (FSK), a cAMP-raising agent (5). As for GLP-1, FSK elicited a step-change in [ATP/ADP]_cyto_ (ΔATP/ADP = 0.09 ± 0.02, n = 13 recordings), although this was notable by the absence of oscillations, most likely due to global activation of soluble ACs (Figure 3, G and H).

### Calcium-dependency of GLP-1-evoked ATP increases

**Figure 4.**
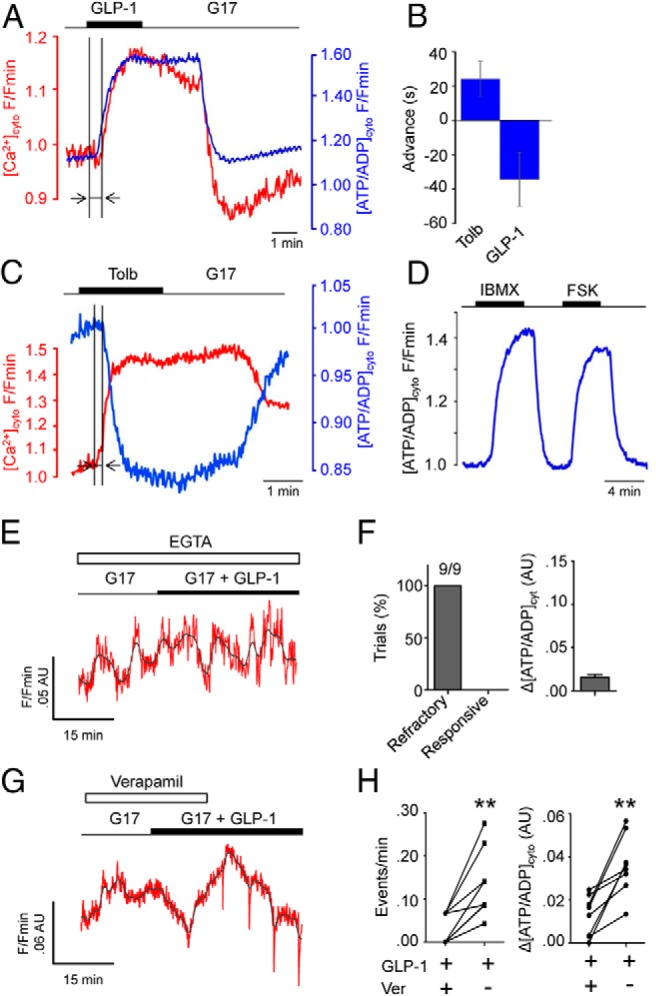
GLP-1 alters beta cell metabolism to initiate the Ca^2+^ influx required to support further [ATP/ADP]_cyto_ increases. A, Simultaneous recordings of Perceval (blue) and Fura-Red (red) in single beta cells reveal that GLP-1-stimulated ATP rises precede those of Ca^2+^ influx. B, Summary bar graph showing the advancement of [ATP/ADP]_cyto_ responses relative to those of Ca^2+^ when cells are stimulated with GLP-1 (measured period is shown with arrows on A and B) (n = 5 recordings). C, As for A, but 200 μM tolbutamide (Tolb) to stimulate large increases in Ca^2+^ and ensuing ATP consuming processes (n = 5 recordings). D, Representative traces showing effects of 10 μM forskolin (FSK) and 100 μM isobutylmethylxanthine (IBMX) on [ATP/ADP]_cyto_ in dissociated beta cells (n = 5 recordings). E, Extracellular Ca^2+^ chelation using EGTA suppresses ATP-responses to GLP-1 (representative trace from n = 11 recordings; red, smoothed; gray, raw). F, EGTA blocked GLP-1 actions in all experiments conducted (9/9). G, As for E, but in the presence of 10 μM of the L-type VDCC blocker verapamil. H, Verapamil (Ver) abolishes GLP-1-induced [ATP/ADP]_cyto_ dynamics and rises (**, *P* < .01 vs GLP-1 + verapamil, Student paired *t* test, n = 11 recordings).

Because GLP-1-induced ATP increases may depend upon or provoke intracellular Ca^2+^ rises due to effects on K_ATP_, dual imaging experiments were performed in isolated beta cells. This preparation was used to minimize photobleaching and interference between signals derived from the two probes that complicated measurements in intact islets. Multiparametric recordings using Perceval and Fura Red revealed that the onset of the ATP/ADP response to GLP-1 preceded any increases in cytosolic Ca^2+^ (Figure 4, A and B and Supplemental Figure 1), indicating that initiation of the latter is likely to be a downstream consequence rather than the cause of incretin-induced [ATP/ADP]_cyto,_ increases. In this case, tolbutamide was used as a control to stimulate large Ca^2+^ rises, which precede net ATP/ADP consumption (Figure 4C). As for experiments using intact islets, FSK was able to evoke increases in ATP in dissociated cells and this could also be mimicked using the phosphodiesterase inhibitor isobutyl methyl xanthine (IBMX) (Figure 4D).

Mitochondrial Ca^2+^ sequestration stimulates citrate cycle dehydrogenases (20, 37) to increase ATP production. Therefore, the effects of extracellular Ca^2+^ chelation were examined to delineate whether continued Ca^2+^ influx through VDCCs was required for the actions of GLP-1 on [ATP/ADP]_cyto_ at elevated glucose concentration, or whether intracellular pathways were relatively more important. Islets perifused with buffer containing zero added Ca^2+^ plus 1 mM ethylene glycol tetraacetic acid (EGTA) failed to display any changes in [ATP/ADP]_cyto_ following application of GLP-1 (Figure 4, E and F). Because the removal of external Ca^2+^ may lead to depletion of internal Ca^2+^ stores or islet dissociation, Ca^2+^ influx through L-type VDCC was blocked using 10 μM verapamil. In line with the above observations, verapamil inhibited the effects of GLP-1 on ATP dynamics (0.03 ± 0.01 vs 0.14 ± 0.02 events/minutes, during and after verapamil, respectively; *n* = 9 recordings, *P* < .01) (Figure 4, G and H).

### GLP-1 modulates ATP dynamics in a species-specific manner

**Figure 5.**
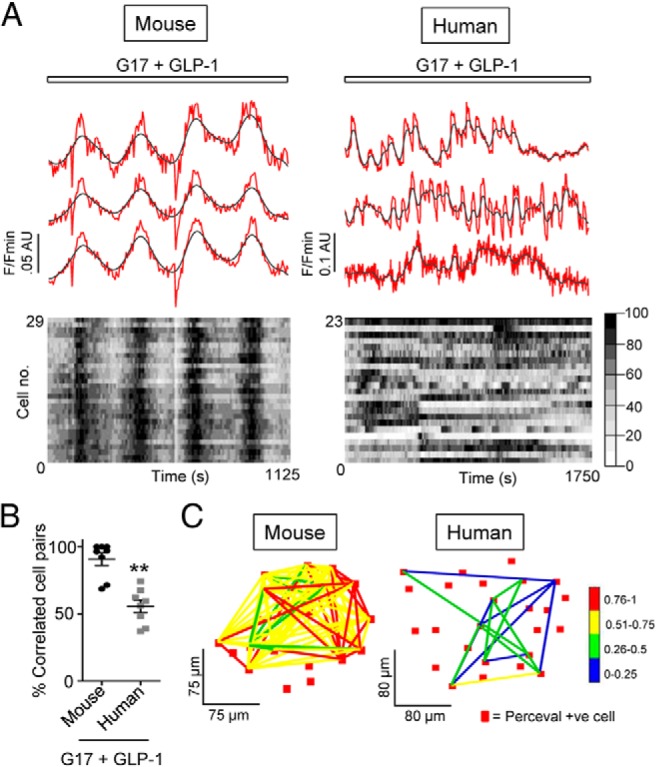
GLP-1-regulated [ATP/ADP]_cyto_ dynamics are species-specific. A, Top panel: GLP-1 induces highly synchronized [ATP/ADP]_cyto_ oscillations in mouse islets (representative traces from three individual beta cells). Although GLP-1 induces similar ATP rises in human islets (68), population dynamics are largely stochastic. Bottom panel: heatmap depicting min-max (0–100%) for each cell in grayscale. B, Mean percentage significantly correlated cell pairs is lower in GLP-1-treated human vs mouse islets (**, *P* < .01 vs mouse; Mann-Whitney *U* test) (n = 8 recordings from three donors and four animals). C, Representative connectivity map displaying the location (x-y) of significantly correlated cell pairs from the GLP-1-responsive beta cell population. Note the relative paucity and weakness of correlated links in human vs mouse islets (*P* < .05) (correlation strength is color-coded; 0 [blue] = lowest, 1 [red] = highest).

We have recently shown that incretins augment insulin secretion in human islets by boosting beta cell cooperativity in a process termed “incretin-regulated cell connectivity” (17). Given that similar effects are largely absent in mouse islets, we wondered whether incretin could differentially affect ATP/ADP dynamics to influence the metabolism thought to drive ionic oscillations (38, 39). To investigate this, large-scale mapping of cell-cell correlations was used to determine the effects of GLP-1 on the population organization of metabolic oscillations in mouse and human tissue over a 30–40-minute period (17, 40). During GLP-1 application, the responsive subpopulation within mouse islets mounted synchronous deflections in [ATP/ADP]_cyto_ (Figure 5A). By contrast, human islets responded to the same challenge with largely asynchronous ATP oscillations (Figure 5A). In line with this, correlated activity was much higher in mouse compared with human islets (Figure 5B), as evidenced by lower levels of metabolic connectivity in the latter species (90.7 ± 4.6 vs 55.6 ± 4.7% significantly correlated cell pairs, mouse vs human tissue, respectively; n = 8 recordings from three donors and four animals, *P* < .01) (Figure 5C).

## Discussion

**Figure 6.**
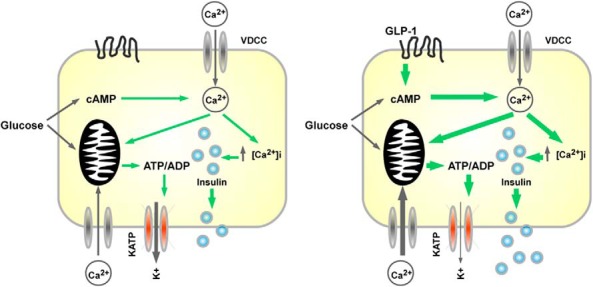
Schematic of GLP-1-modulated beta cell energetics. Glycolytic metabolism of glucose stimulates insulin secretion through increases in [ATP/ADP]_cyto_ and cAMP, leading to opening of VDCC and Ca^2+^ influx, the latter reinforcing ATP synthesis. GLP-1 augments glucose-stimulated insulin secretion by increasing cAMP input, leading to potentiated [ATP/ADP]_cyto_ rises and Ca^2+^ influx.

The literature surrounding the action of incretin on beta cell ATP/ADP ratios is contentious, with the existence of conflicting reports regarding the effects of the incretins upon cellular metabolism and energetics (15, 16). By combining recombinant expression of the ATP sensor Perceval with in situ imaging of ATP/ADP dynamics in intact mouse islets, we demonstrate that GLP-1 likely influences beta cell intermediary metabolism at both low and high glucose concentrations. Mechanistically, these effects involved changes in mitochondrial potential, GLP-1R engagement, and ATP-triggered Ca^2+^ influx through VDCC, and could be mimicked using the cAMP-elevating compounds FSK or IBMX (see Figure 6 for a schematic).

Our observations with Perceval reflect steady-state [ATP/ADP]_cyto_ and, as such, report the balance between the rates of ATP synthesis and degradation. However, GLP-1 seems unlikely to inhibit ATP-consuming processes under the conditions used here because it acutely acts to enhance ionic fluxes and insulin secretion (17). Thus, increases in [ATP/ADP]_cyto_ in response to the incretin seem more likely to reflect accelerated ATP synthesis. However, we cannot formally exclude the possibility that factors other than enhanced metabolism, such as a redistribution of ATP between different intracellular pools (eg, secretory granules or the endoplasmic reticulum) (41), contribute to the observed actions of GLP-1 on the cytosolic ATP/ADP ratio.

In response to elevated glucose, and extending our earlier findings, which used the less sensitive photoprotein firefly luciferase to image cytosolic and mitochondrial-free ATP in islets (19), mouse beta cells responded by mounting oscillations in ATP/ADP that were coordinated across the imaged population. This may reflect a bidirectional interplay between metabolic and ionic signals that is phase-set by negative feedback emanating at the level of Ca^2+^, and which drives the slow oscillations in K_ATP_ conductance and Ca^2+^ influx (21, 39). Notably, only ∼50% of the Perceval-expressing population displayed [ATP/ADP]_cyto_ rises/oscillations following exposure to high glucose, raising the intriguing possibility that a metabolically coupled subnetwork of beta cells orchestrates the global Ca^2+^ dynamics known to underlie insulin secretion in mouse islets (31, 42). Although electrotonic coupling via gap junctions would allow nonmetabolically active beta cells to contribute to islet-wide Ca^2+^ oscillations (31, 43), previous studies have shown that nicotinamide adenine dinucleotide phosphate (NAD(P)H) increases are observed in 90% of beta cells within islets (44). Potential explanations for these discrepancies include the existence of functional heterogeneity between beta cell subpopulations including differences in ATP generation (45), compartmentalization of ATP responses into discrete domains (eg, in the subplasma membrane space) that remain undetectable at the resolutions employed here (21), and probe saturation returning values under the 20% threshold for inclusion as responsive cells.

Of note, GLP-1 was able to influence ATP/ADP dynamics within a matter of minutes, principally by altering both levels and patterning, and the former effect was confirmed using static biochemical assays. The significance of the reported changes to ATP dynamics remains unknown. Given that GLP-1 increases intracellular Ca^2+^ load (17), the pronounced and rapid downward deflections in [ATP/ADP]_cyto_ may reflect either Ca^2+^-dependent ATP-consuming processes (eg, exocytosis, Ca^2+^ store refilling, etc.), or alternatively, a mechanism to prevent mitochondrial Ca^2+^ toxicity, both of which are rapidly balanced by augmented metabolism. In terms of the latter mechanism, the uptake of ATP into the mitochondrial matrix via the Ca^2+^-activated ATP-Mg/Pi transporter would buffer intramitochondrial [Ca^2+^], preventing mitochondrial permeability transition and cell death at the expense of [ATP/ADP]_cyto_.

Although mitochondrial Ca^2+^ uptake stimulates NADH production to drive respiratory chain activity and ATP synthesis (37), an exaggerated flux of Ca^2+^ across the inner mitochondrial membrane can constrain ATP production by reducing the electrochemical gradient that powers proton pumping and F_1_/F_0_ ATP-synthase activity. We therefore used TMRE to monitor whether GLP-1 was able to alter ψ_m_ to evoke [ATP/ADP]_cyto_ rises. Whereas glucose exerted a rapid and large hyperpolarizing influence upon ψ_m_, as previously reported (26), this was much less pronounced in incretin-stimulated islets. The relatively larger increases in ATP detected in response to GLP-1 per unit decrease in ψ_m_ is consistent with a higher rate of Ca^2+^ entry and ATP consumption in response to glucose than incretin (eg, to drive increased secretory granule dynamics, ion pumping, and protein synthesis).

In line with our previous reports using MIN6 beta cells (15), GLP-1 was able to modulate ATP/ADP dynamics even under conditions of low glucose. Although changes in [ATP/ADP]_cyto_ would be expected to close hyperpolarizing K_ATP_ channels, leading to depolarization and Ca^2+^ influx, GLP-1 was unable to increase cytosolic free Ca^2+^ in beta cells exposed to nonpermissive glucose concentration. Thus, other glucose-derived signals may be required to translate GLP-1-induced oscillations in [ATP/ADP]_cyto_ into Ca^2+^ rises and Ca^2+^-dependent insulin secretion. Indeed, glucose and GLP-1 engage distinct ACs (46), and summation and/or cell compartmentalization of the ensuing changes to cAMP-Epac dynamics may therefore be required to fully sensitize K_ATP_ to GLP-1-stimulated alterations to beta cell metabolism (47, 48). Although the mechanisms underlying GLP-1 effects at low glucose remain unknown, they may implicate a role for mitochondrial ψ_m_ that was altered by the incretin even in the presence of nonpermissive levels of the sugar.

Simultaneous recordings of Perceval and Fura Red revealed that GLP-1 elicited rises in [ATP/ADP]_cyto_ before those of [Ca^2+^]_i_, supporting the notion that metabolism and K_ATP_ are required to initiate GLP-1-stimulated Ca^2+^ influx. It is worthwhile to note that the response time for Perceval (seconds) is much slower than that for Fura Red (milliseconds) (23, 49), meaning that the lag between the onset of [ATP/ADP]_cyto_ and Ca^2+^ increases was likely underestimated. Continued effects of GLP-1 upon beta cell metabolism were dependent upon Ca^2+^ influx through L-type VDCCs, because the incretin was unable to stimulate [ATP/ADP]_cyto_ rises in cells pretreated with EGTA and verapamil. This is unsurprising given our recent findings that mitochondrial Ca^2+^ uptake through the mitochondrial Ca^2+^ uniporter is critical for rendering beta cells glucose competent (24, 26). This effect is most likely achieved through the Ca^2+^-stimulated up-regulation of mitochondrial dehydrogenase activity, which supplies reducing equivalents to the respiratory chain, leading to enhanced ATP production (50, 51). Although it could be argued that the observed effects were due to blockade of glucose actions, which then reappeared following washout, it should be noted that GLP-1 was unable to alter [ATP/ADP]_cyto_ during antagonist application, making an effect of the incretin on beta cell metabolism via a nonextracellular Ca^2+^-linked pathway unlikely. We observed that, following washout of both verapamil and exendin 9-39, the increases in [ATP/ADP]_cyto_ were smaller than those observed under control conditions; this may reflect residual blockade of GLP-1R/VDCC or alternatively slow dissociation kinetics due to use of a perfusion system.

A key observation here (Figure 4B) was that the onset of GLP-1-induced increases in [ATP/ADP]_cyto_ occurred before detectable changes in cytosolic free Ca^2+^. What, therefore, may be the mechanisms through which GLP-1 leads to an apparent direct stimulation of ATP synthesis? A recent report (52) has suggested that the GLP-1 receptor agonist geniposide may stimulate pyruvate carboxylase activity in beta cells to promote anaplerosis into the citrate cycle and hence ATP synthesis (53). These observations are in line with the present findings and provide one possible mechanism through which elevated cAMP may enhance ATP production. Other possibilities include stimulation of glucokinase (54), enhanced protein kinase A–dependent breakdown of fuel stores such as glycogen or triglycerides (55) and, conceivably, activation of glycolytic enzymes including phosphofructokinase (56). Finally we note, intriguingly, that intramitochondrial cAMP levels may fluctuate independently of those in the cytosol (57) to regulate intramitochondrial ATP synthesis, though whether GLP-1 is able to engage this pathway is unclear given the apparent impermeability of the mitochondrial inner membrane to cAMP.

We have recently shown that human islets mount poorly coordinated Ca^2+^ responses to glucose, but high levels of correlated activity between beta cells can be driven by GLP-1 in a process termed “incretin-regulated connectivity.” By contrast, mouse islets already display high levels of coordinated activity in response to glucose, and GLP-1 acts to increase time spent in the active state while maintaining synchronicity within the beta cell syncytium (17, 58). We therefore sought to clarify whether these species differences in incretin potentiation of glucose-stimulated insulin secretion were accompanied by alterations to metabolism. Interestingly, whereas GLP-1-induced [ATP/ADP]_cyto_ dynamics were highly correlated across the responsive beta cell subpopulation in mice, they were more stochastic in human islets. Thus, GLP-1 is a poor orchestrator of metabolic communications between human beta cells, and such uncoupling of metabolic and ionic oscillations may contribute to species-specific regulation of the incretin axis. Although the mechanisms remain unexplored, they may reflect divergent islet architecture, paracrine/autocrine signaling circuits, and gap junction function in human vs mouse tissue (58, 59). We cannot exclude, however, a role for species-differences in beta cell physiology introduced by islet isolation procedures, cold ischemia time, the relative immaturity of mouse compared with human islets, and the more varied nature of human islet material in terms of sex, age, and body mass index (see references (60, 61) for useful discussion).

Finally, although we refer to measurement of [ATP/ADP]_cyto_ throughout the present study, in the case of Perceval, this requires ADP levels commensurate with probe affinity (23). Because calculation of free ATP and ADP cannot readily be inferred from total levels due to nucleotide sequestration in organelles and cytoskeleton binding (41, 62), the actual parameter under measure may vary. Similarly, our own measurements of total ATP, while most easily explained through changes in phosphorylation potential and ATP/ADP ratio (63), could conceivably reflect a change in the concentration of ATP alone. Future studies using probes with a range of affinity values will therefore be required to accurately quantify the effects of glucose and incretin on intracellular [ATP/ADP]_cyto_.

In summary, we show that GLP-1 is able to influence beta cell intermediary metabolism through alterations to the extent and patterning of intracellular ATP/ADP increases. This requires GLP-1R signaling, changes to ψ_m_ and extracellular Ca^2+^ influx, and displays marked species divergence in “metabolic connectivity,” as human beta cells fail to properly coordinate [ATP/ADP]_cyto_ oscillations. Thus, alterations to beta cell metabolism may contribute to the diverse and glucose-dependent actions of incretin, including potentiation of insulin secretion and prevention of apoptosis.

## Materials and Methods

### Mouse islet isolation

Animals were maintained in individually ventilated cages in a specific pathogen–free facility under a 12-h light-dark cycle with ad libitum access to water and food. Mice (8–12 weeks old) were euthanized by cervical dislocation and pancreatic islets isolated by collagenase digestion, as described (64). Animal procedures were approved by the Home Office according to the Animals (Scientific Procedures) Act 1986 of the United Kingdom (PPL 70/7349).

### Human islet isolation

Human islets (donor age range, 34–52 years) were isolated at transplantation facilities in Oxford, Geneva, and Pisa with the relevant national and local ethical permissions, including consent from next of kin where required. Islets were cultured as previously described (17). All studies involving human tissue were approved by the National Research Ethics Committee London (Fulham) “Signal Transduction in isolated human islets: regulation by glucose and other stimuli” REC No. 07/H0711/114.

### Adenoviral delivery of Perceval

Complementary DNA encoding the ATP/ADP sensor Perceval (a kind gift from Professor Gary Yellen) was cloned and packaged into adenovirus as described (25, 26). Forty-eight-hour incubation with virus was sufficient to express Perceval throughout the first few islet cell layers.

### Immunohistochemistry

Islets were fixed overnight at 4 C in paraformaldehyde (4%, wt/vol) before application of guinea pig anti-insulin 1:200 and mouse anti-glucagon 1:1000 antibodies, and processing as previously described (17). Uniform linear adjustments were applied to contrast/brightness to improve image quality for presentation.

### ATP/ADP and Ca^2+^ imaging

Perceval-expressing islets were placed in a custom-manufactured 36 C chamber (Digital Pixel) and perifused with a HEPES-bicarbonate buffer (120 mM NaCl, 4.8 mM KCl, 24 mM NaHCO_3_, 0.5 mM Na_2_HPO_4_, 5 mM HEPES, 2.5 mM CaCl_2_, and 1.2 mM MgCl_2_) saturated with 95% O_2_/5% CO_2_ and adjusted to pH 7.4. A solid-state 491-nm laser was passed through a Nipkow spinning-disk head (Yokogawa CSU-10) coupled to ×10–×20/0.3–0.5NA objectives (EC Plan-Neofluar, Zeiss). Emitted signals (510–540 nm) were subsequently captured at a frame rate of 0.2 Hz using a highly-sensitive 16-bit 512×512-pixel electron multiplying charge-coupled device (Hamamatsu). Perceval-expressing cells were manually delineated using a region of interest and intensity over time traces extracted. Signals were normalized using the function F/Fmin where F is fluorescence at a given time point and Fmin is the minimum recorded fluorescence.

Multiparametric recordings of [ATP/ADP]_cyto_ and [Ca^2+^]_i_ were performed as previously detailed (25, 26). Briefly, Perceval-expressing cells were loaded with Fura-Red and both fluorophore emissions acquired at 0.2 Hz using an Olympus IX-71 microscope equipped with a UPlanFL N ×40/1.3NA objective. The excitation/emission wavelengths were (nm): 490/630 (Fura-Red) and 490/535 (Perceval). GLP-1-induced pH changes were determined using the ratiometric dye 2′,7′-Bis(2-carboxyethyl)-5(6)-carboxyfluorescein.

### Biochemical detection of ATP

Batches of 10 islets were treated as indicated for 30 minutes before removal of supernatant and extraction of lysate using either percholoric acid or distilled boiling H_2_O followed by sonication and storage on ice (65, 66). ATP concentration in the supernatant was immediately assayed using a luciferase-based detection kit according to the manufacturer's instructions (ATP Determination Kit, Life Technologies).

### TMRE imaging

Islets were incubated in 20 nM TMRE for 30 minutes before imaging as above, but using excitation and emission wavelengths of 563 nm and 600 nm, respectively. Treatments were applied as indicated and at the end of each recording, carbonyl cyanide 4-(trifluoromethoxy)phenylhydrazone was added at 2 μM.

### Correlation and wavelet analyses

Correlation analyses were performed using the Pearson r coefficient as previously detailed (*P* < .05) (17, 67). Phase maps were compiled by converting the normalized intensity of each cell to a value between 1–100% and assigning this to a color. To depict the contribution of period to [ATP/ADP]_cyto_ dynamics, the frequency and time components of the mean Perceval fluorescence trace were extracted using bias-corrected wavelet analysis.

### Statistical analysis

Data distribution was determined using the D'Agostino omnibus test. Pairwise comparisons were performed using Mann-Whitney *U* test or Student unpaired and paired *t* tests. Interactions between multiple treatments were assessed using one-way ANOVA followed by Bonferonni's post hoc test. A sigmoidal fit was used to calculate the EC50 of normalized and log-transformed dose-response curves. Analyses were conducted using R (R-project), Graphpad Prism (Graphpad Software) and IgorPro (Wavemetrics), and results deemed significant at *P* < .05.

## Additional material

Supplementary data supplied by authors.
