# Supplementary material for: Incretin-Modulated Beta Cell Energetics in Intact Islets of Langerhans
Source: Mol Endocrinol. 2014 Apr 25;28(6):860–71. doi: 10.1210/me.2014-1038 (PMC4042069; doi:10.1210/me.2014-1038)
Supplement: Supplementary file 1 [file me-14-1038.pdf]

Supplementary Figure 1: Simultaneous monitoring of Ca^{2+} and ATP in single mouse beta cells. **(A)** Representative recording demonstrating that GLP-1 stimulates ATP rises before those of Ca^{2+} (magnification inset) (*n* = 5 recordings). **(C)** As for (A) but tolbutamide as a control to stimulate large excursions in Ca^{2+} which lead to net ATP consumption (magnification inset) (*n* = 5 recordings).

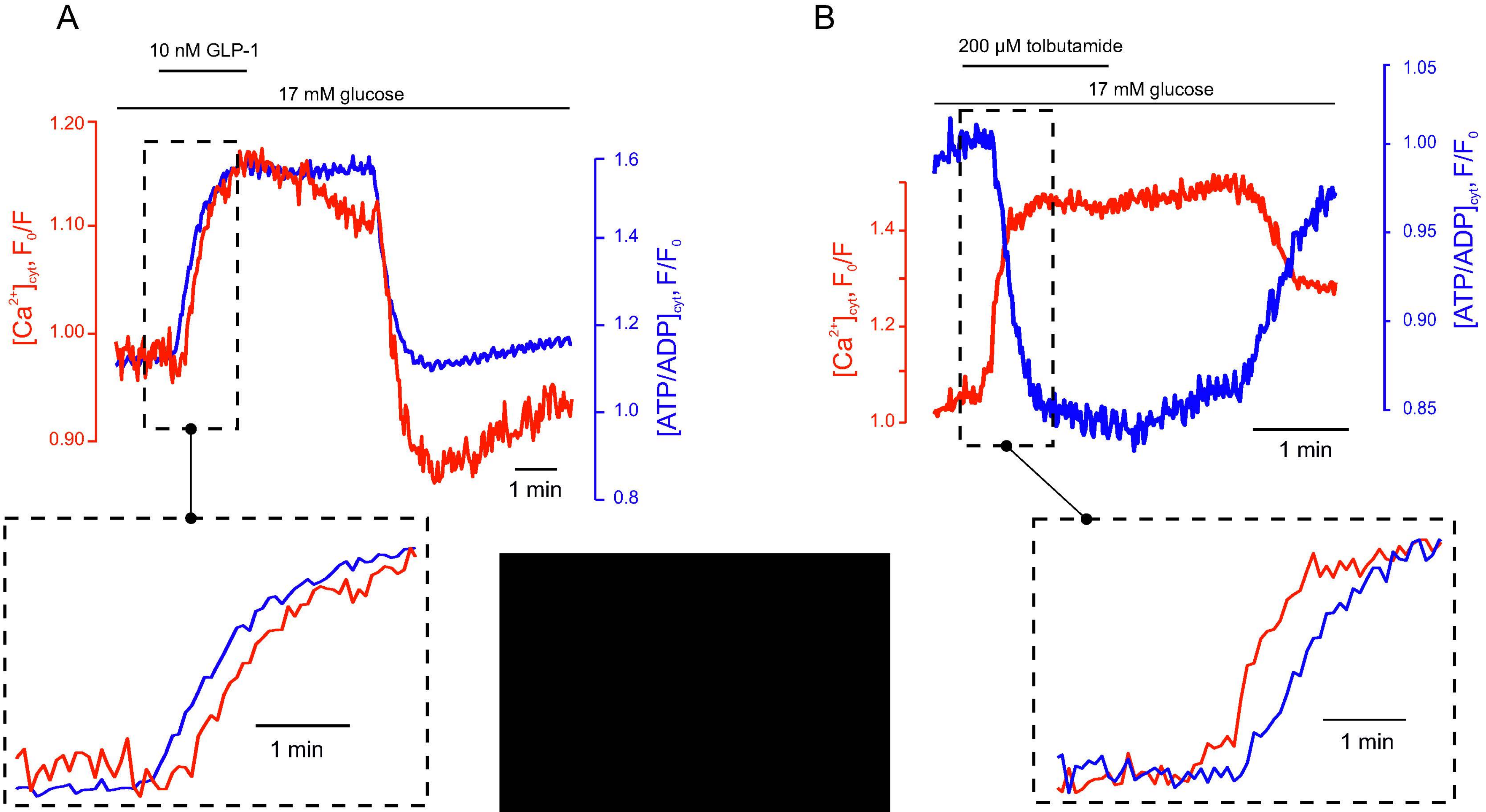

A
10 nM GLP-1
17 mM glucose
$[Ca^{2+}]_{cyt}$, F_0/F
1.20
1.10
1.00
0.90
$[ATP/ADP]_{cyt}$, F/F_0
1.6
1.4
1.2
1.0
0.8
1 min
1 min
B
200 µM tolbutamide
17 mM glucose
$[Ca^{2+}]_{cyt}$, F_0/F
1.4
1.2
1.0
$[ATP/ADP]_{cyt}$, F/F_0
1.05
1.00
0.95
0.90
0.85
1 min
1 min
